# TiO_2_-Decorated by Nano-γ-Fe_2_O_3_ as a Catalyst for Efficient Photocatalytic Degradation of Orange G Dye under Eco-friendly White LED Irradiation

**DOI:** 10.1021/acsomega.3c06420

**Published:** 2023-10-13

**Authors:** Ahmed Halfadji, Abdelkader Chougui, Rania Djeradi, Fatima Zohra Ouabad, Hanane Aoudia, Shashanka Rajendrachari

**Affiliations:** † Synthesis and Catalysis Laboratory, Ibn Khaldoun University of Tiaret, Tiaret 14000, Algeria; ‡ Department of Sciences and Technology, Faculty of Applied Sciences, Ibn Khaldoun University of Tiaret, Tiaret 14000, Algeria; § Department of Chemistry, Ibn Khaldoun University of Tiaret, Tiaret 14000, Algeria; ∥ Department of Metallurgical and Materials Engineering, Bartin University, Bartin 74100, Turkey

## Abstract

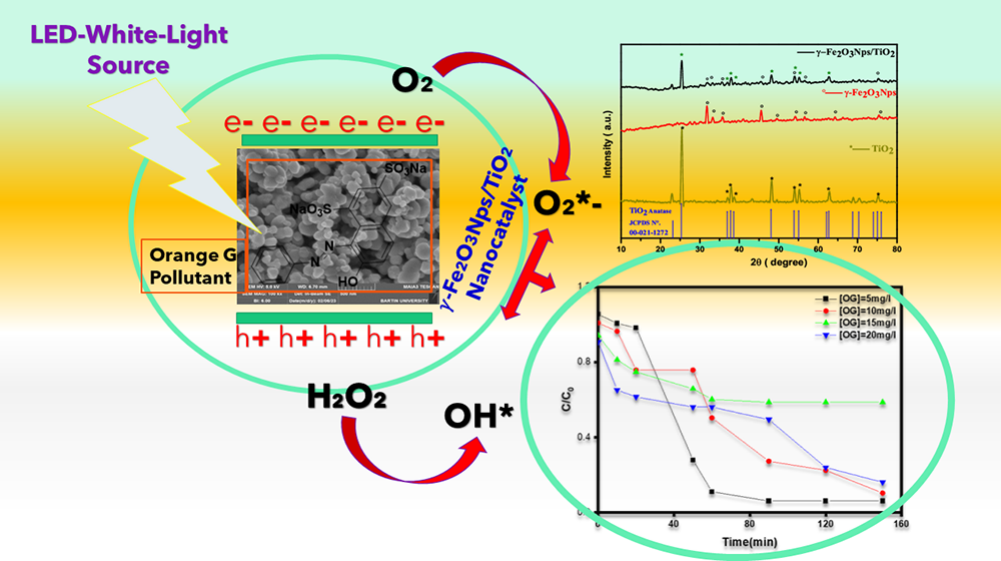

Azo dyes make up a major class of dyes that have been widely studied for their diverse applications. In this study, we successfully applied nano-γ-Fe_2_O_3_/TiO_2_ as a nanocatalyst to improve the photodegradation efficiency of azo dyes (Orange G (OG) dye as a model) from aqueous solution under white light-emitting diode (LED) irradiation. We also investigated the degradation mechanisms and pathways of OG dye as well as the effects of the initial pH value, amount of H_2_O_2_, catalyst dosage, and dye concentration on the degradation processes. The characterizations of nano-γ-Fe_2_O_3_ and γ-Fe_2_O_3_ Nps/TiO_2_ were carried out using various techniques, including X-ray diffractometry, scanning electron microscopy, energy-dispersive X-ray spectroscopy, Fourier transform infrared spectroscopy, and UV–visible spectroscopy. The efficiency of the photodegradation reaction of OG was found to follow pseudo-first-order kinetics (Langmuir–Hinshelwood model) with a rate constant of 0.0338 min^–1^ and an *R*^2^ of 0.9906. Scavenger experiments revealed that hydroxyl radicals and superoxide anion radicals were the dominant species in the OG photocatalytic oxidation mechanism. This work provides a new method for designing highly efficient heterostructure-based photocatalysts (γ-Fe_2_O_3_ Nps/TiO_2_) based on LED light irradiation for environmental applications.

## 1 Introduction

Environmental contamination is largely caused by synthesized organic contaminants, many of which find their way into wastewater. Pesticides, polynuclear aromatic hydrocarbons (PAHs), polychlorinated biphenyls (PCBs), halogenated aliphatic hydrocarbons, halogenated ethers, monocyclic aromatics, pharmaceutical, personal care products, and dyes all pose a threat to the environment (water, groundwater, wastewater, soil, and air).^1,2^ The majority of these pollutants are produced by industrial sources, such as refineries, organic chemical producers, steel mills, coal conversion, and textile mills.^2^ A problem currently faced by developed and underdeveloped countries around the world is colored wastewater from the textile industry. Wastewater from textile and dye industries is highly colored with a significant amount of auxiliary chemicals (synthetic dyes). These compounds can transform into toxic and carcinogenic compounds upon release in natural environments, mainly in aqueous media.^3^ Azo dyes represent the largest group of dyes and are characterized mostly by aromatic moieties and are bonded together by −N=N– bonds (a type of chromophore). Additionally, azo dyes with their complex and steady chemical structures make them resistant to biodegradation or chemical degradation; thus, traditional physical, chemical, and biological treatment methods are ineffective and costly for removal from water. Also, it can cause problems when treated sewage water is reintroduced into natural waterways. Organic contaminants are known or suspected to be carcinogens or mutagens, which can have adverse effects on aquatic life and drinking water quality. In 1890, Fenton discovered a homogeneous catalytic oxidation process (Fenton process) using hydrogen peroxide (H_2_O_2_) and ferrous ions (Fe^2+^) in an acidic medium,^4^ while the photo-Fenton process occurs under a source of irradiation. The photo-Fenton reaction is the most efficient, cost-effective, and advanced oxidation process for treating nonbiodegradable organic pollutants in water.^5^ The Fenton oxidation process produces hydroxide radicals, HO·, by reacting H_2_O_2_ with Fe^3+^/Fe^2+^ ions as a catalyst, in the following mechanism reactions (eqs 1–7)^5^:
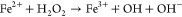
1
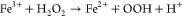
2
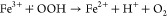
3
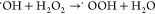
4
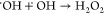
5
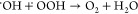
6
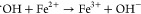
7

Numerous studies have been conducted on using Fenton oxidation for the treatment of azo dye wastewater,^6^ in which ·OH’s concentration in the process and the generation rate played a key role in the decolorization efficiency of several azo dyes degraded by Fenton oxidation.^7^ Also, recent studies have reported the efficiency of photo-Fenton oxidation using TiO_2_, CdS, and WO_3_ coated and mixed with iron oxide.^8^ Using modified TiO_2_, Fe^3+^, and H_2_O_2_, we can significantly enhance the production of ·OH and the degradation of synthetic dyes and other organic pollutants.^9^ On the other hand, to perform the conventional photo-Fenton reaction, a Fenton-like reaction by mixing TiO_2_ and Fe^3+^/Fe^2+^ as catalysts has been developed.^10^ Several studies have reported that these TiO_2_/Fe_2_O_3_ nanocomposites as catalysts are effective in removing nonbiodegradable contaminants, such as dyes and antibiotics from wastewater.^11^ This is caused by an increase in the catalyst surface area, which leads to catalyst activity depletion.^10^ Most recently, TiO_2_/γ-Fe_2_O_3_ nanocomposites have been developed that have the highest catalytic efficiency and stability for the degradation and removal of organic pollutants such as ciprofloxacin (CIP),^12^ metronidazole (MNZ),^13^ ibuprofen,^14^ Auramine (AM) dye,^15^ bisphenol A (BPA),^16^ and rhodamine B (RhB) dye.^17^

Prior to discovering light-emitting diode (LED) lighting, the existing luminaires consumed a substantial amount of energy but offered subpar luminescence efficiency. LED lighting stands out due to its extended operational lifespan and ecological characteristics, as it contains no hazardous mercury.^18^ In addition to energy conservation, LEDs possess the unique capacity for meticulous tuning and control; for example, the emission spectrum could be optimized for our health and well-being.^19^ An LED is typically made from semiconductors containing inorganic phosphors along with specific encapsulating materials. Several semiconductor compounds are commonly used to produce LEDs, including gallium nitride (GaN), aluminum gallium nitride (AlGaN), indium gallium nitride (InGaN), aluminum indium gallium phosphide (AlInGaP), gallium arsenide (GaAs), and aluminum gallium arsenide (AlGaAs).^20^ Different LEDs are used for a range of applications, including curing chemicals or polymers, analyzing data in laboratories, and even disinfecting water and medical equipment. Recently, photocatalysis-activated composites have been used to degrade persistent compounds in the presence of visible LED light.^21^ Most commercial LEDs contain InGaN, which can produce near-UV, violet, blue, or green light within a wavelength range of 395 to 530 nm.^22^

Many studies have recently been interested in applying solar light to photocatalytic degradation of organic pollutants, but they have no alternative illumination source in the absence of solar light (during the night period); for this reason, this study aimed to (i) synthesize and characterize of γ-Fe_2_O_3_ nanoparticles (Nps) and γ-Fe_2_O_3_ Np/TiO_2_ nanocomposites, (ii) evaluate the photocatalytic activity of the prepared γ-Fe_2_O_3_ Nps/TiO_2_ for decolorization of Orange G (OG) azo dye by photo-Fenton oxidation under LED illumination, and (iii) study the effects of various operation conditions such as pH of the solution, the dosage of H_2_O_2_, the dosage of the catalyst, and the level of contamination on the removal of OG in aqueous solution under LED irradiation. (iv) Kinetic degradation and scavenger experiments were conducted to identify oxidizing species, which led to hypotheses about the underlying reaction mechanism of photo-Fenton oxidation.

## 2 Materials and Methods

### 2.1 Materials

**Figure 1:**
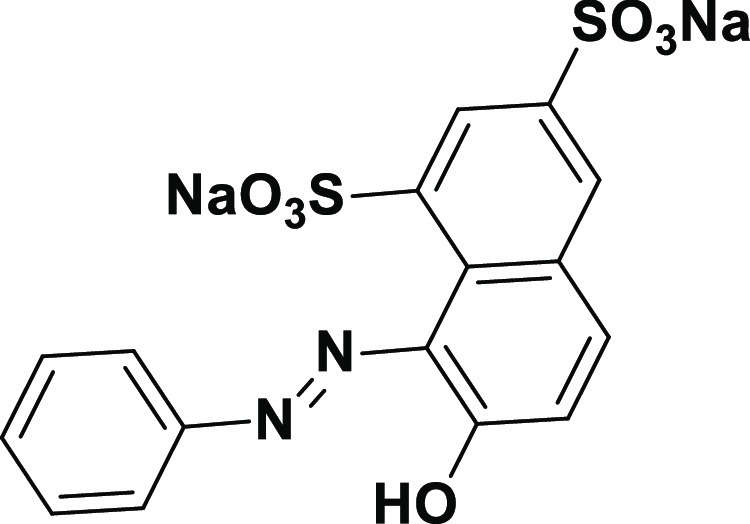
Chemical structure of Orange G.

The organic pollutant OG (7-hydroxy-8-[(*E*)-phenyldiazenyl] naphthalene-1,3-disulfonic acid, C_16_H_10_N_2_Na_2_O_7_S_2_, azo dye) (Figure 1) and titanium dioxide (TiO_2_) were purchased from Biochem, Germany, with purity >98%. To prepare γ-Fe_2_O_3_, iron(II) chloride (FeCl_2_·4H_2_O, 99%) and iron(III) chloride (FeCl_3_, 97%) were purchased from Fluka and Sigma-Aldrich, Spain. Sodium hydroxide (NaOH, 98%) and hydrogen chloride (HCl, 37%) were obtained from Panreac, Germany. *tert*-Butanol (TBA), benzoquinone (BQ), and ethylenediaminetetraacetic acid disodium salt (EDTA-2Na) were purchased from Sigma-Aldrich. Hydrogen peroxide (H_2_O_2_, 30%) was purchased from SialChim Reagent Ltd., and ethanol at 96.3% in analytical grade was obtained from VWR Chemicals. Experimental solutions were prepared with doubly distilled water, and all reagents were used without further purification.

### 2.2 Synthesis of γ-Fe_2_O_3_ Nps

γ-Fe_2_O_3_ Nps are prepared via the simple chemical coprecipitation method. In brief, under magnetic stirring, 1.718 mg of iron(II) chloride, FeCl_2_·4H_2_O, and 4.66 mg of iron(III) chloride, FeCl_3_, were dissolved in distilled water (100 mL). All experiments were conducted with a mole ratio of 1:2 between iron(II) chloride (0.0864 M) and iron(III) chloride (0.175). The solution was mixed at room temperature with magnetic stirring. In the mixing step, ammonium hydroxide (10 M) was dropped into the solution with vigorous stirring until pH = 10. The obtained dark brown precipitation was dried at 70 °C for 24 h in an oven and then was collected and rinsed three times with ionized water and ethanol, respectively. Finally, the magnetite (γ-Fe_2_O_3_) nanopowder was calcined at 300 °C for 2 h to obtain the light brown γ-Fe_2_O_3_ NPs. γ-Fe_2_O_3_ NPs are the resultant product of the reaction between FeCl_2_·4H_2_O and FeCl_3_, according to eqs 8–10^23^:
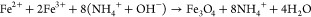
8
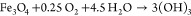
9
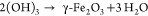
10

### 2.3 Synthesis of γ-Fe_2_O_3_ NPs/TiO_2_

A coprecipitation method was used to synthesize γ-Fe_2_O_3_ Np/TiO_2_ bicomposites, as reported by D’Arcy et al.^24^ Commercial TiO_2_ anatase was coprecipitated with γ-Fe_2_O_3_ Nps in ethanol and then calcined at 400 °C for 6 h. With this process, it is possible to produce nanocomposites with large surface areas. Then, the synthesized nanocomposite γ-Fe_2_O_3_Nps/TiO_2_ was applied as a catalyst to study the photocatalytic degradation of OG from aqueous solution.

### 2.4 Characterization

X-ray diffraction (XRD) patterns of γ-Fe_2_O_3_ Nps and γ-Fe_2_O_3_ Np/TiO_2_ nanocomposites were recorded with a Rigaku MiniFlex 600 X-ray diffractometer with Cu Kα radiation using either a Cu tube operating at 40 kV and 35 mA or a Co tube performing at 35 kV and 30 mA. A scanning rate of 10 °/min was used in a range of 2θ from 10 to 80°. We used these patterns to identify the Ti and Fe phases in mixed oxides by comparison to standard anatase, hematite, and rutile. A Fourier transform infrared (FTIR) spectrophotometer (Shimadzu-8400S) was used to investigate chemical bonding information in the 400–4000 cm wavenumber range. The morphologies and chemical compositions of the samples were analyzed by scanning electron microscopy (SEM) equipped with energy-dispersive spectroscopy (EDS) (SEM–EDS, Tescan-MAIA3 XMU).

### 2.5 Photocatalysis Process

All experiments were conducted in cylindrical batch mode (*D* = 7 cm; *H* = 15 cm; total volume = 100 mL) in a reactor system with continuous stirring to mix and homogenate contact on the solution at room temperature (23–25 °C). The light source was a Philips white LED lamp with 12 W power consumption (emitting 460 and 576 nm ± 10 nm). To prepare the reaction suspension, 100 mL of OG solution was added with the appropriate amounts of the catalyst and H_2_O_2_. To adjust the pH, HCl or NaOH was used, and the measured pH was measured using a pH meter. To reach equilibrium between the catalyst and pollutants, the mixture was mixed in the dark for 30 min before adding H_2_O_2_. After this, 3 mL of suspension was taken using a syringe at given intervals of time and then centrifuged at 4000 rpm for 5 min. Using a UV–vis spectrophotometer, we evaluated the progress of OG degradation by measuring its characteristic absorbance (λ = 478 nm) on solution samples.

Based on the following eq 11, degradation efficiency (*R*%) was calculated as the percent removal of OG by photodegradation.
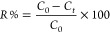
11where *C*_0_ is the initial BM concentration in the solution (mg L^–1^) and *C_t_* is the OG concentration at time (*t*) of the removal process.

## 3 Results and Discussion

In this section, we present and discuss the experimental findings from this study’s work on the synthesis and characterization of nanomaterials (γ-Fe_2_O_3_ and γ-Fe_2_O_3_Nps/TiO_2_) as well as the parameters affecting photocatalytic activity in the degradation of an organic pollutant (OG dye) using γ-Fe_2_O_3_Nps/TiO_2_ under white LED light irradiation (12 W).

### 3.1 Characterization of γ-Fe_2_O_3_ Nps and γ-Fe_2_O_3_ Nps/TiO_2_

#### 3.1.1 UV–Vis Analysis

The diffuse reflectance spectroscopy (DRS) analyses were obtained using a Shimadzu UV–vis–NIR UV 3600 scanning spectrophotometer in a wavelength range of 200–1100 nm.

**Figure 2:**
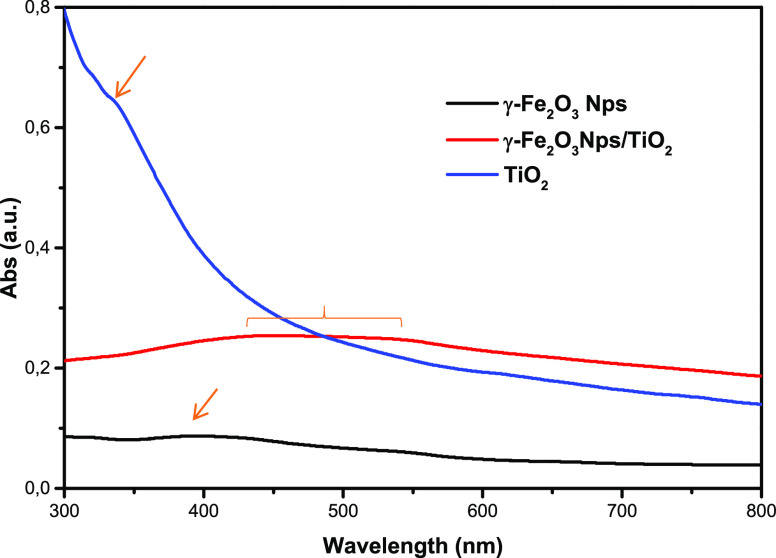
UV–vis absorption spectra of TiO_2_, γ-Fe_2_O_3_ Nps and γ-Fe_2_O_3_ Np/TiO_2_ samples.

UV–vis absorption spectra of γ-Fe_2_O_3_ Nps/TiO_2_, TiO_2_, and γ-Fe_2_O_3_ Nps are shown in Figure 2. UV adsorption region of the TiO_2_ spectrum shows a distinct absorption band edge between 350 and 400 nm, which is linked to photoexcitation from valence to conduction bands. In addition, for γ-γ-Fe_2_O_3_ Np/TiO_2_ and γ-Fe_2_O_3_ Np samples, which are embedded iron oxide, their UV spectra can significantly exhibit a higher wavelength, which was attributed to the band gap excitation of γ-Fe_2_O_3_, whereas the absorbance peak 380–540 nm is due to the band gap absorption of the TiO_2_ phase in the γ-Fe_2_O_3_ Np/TiO_2_ sample.

These findings revealed a significant difference in light absorption between the samples, indicating that they were shifted into visible light after γ-Fe_2_O_3_ was added. The charge transfer transition from oxygen to iron can cause the absorbance to shift to a longer wavelength.^3,24^ As a result, Fe^3+^ incorporation into TiO_2_ in the γ-Fe_2_O_3_ Np/TiO_2_ nanocomposite sample was shown by the UV–vis spectra results.

Tauc’s plot (eq 12) was used to determine the band gap (*E*_g_) of the synthesized γ-Fe_2_O_3_ Np/TiO_2_ nanocomposite, where *h*ν and α*h*ν^2^ were calculated as the horizontal and vertical coordinates, respectively. *E*_g_ can be calculated directly from the intersection of the tangent line and horizontal axis to be 2.25 eV.
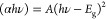
12where α is the absorption coefficient near the absorption edge, *h* is the Planck constant, ν is the frequency of light, and *A* is a band edge constant, *n* = 1.

#### 3.1.2 FTIR Analysis

**Figure 3:**
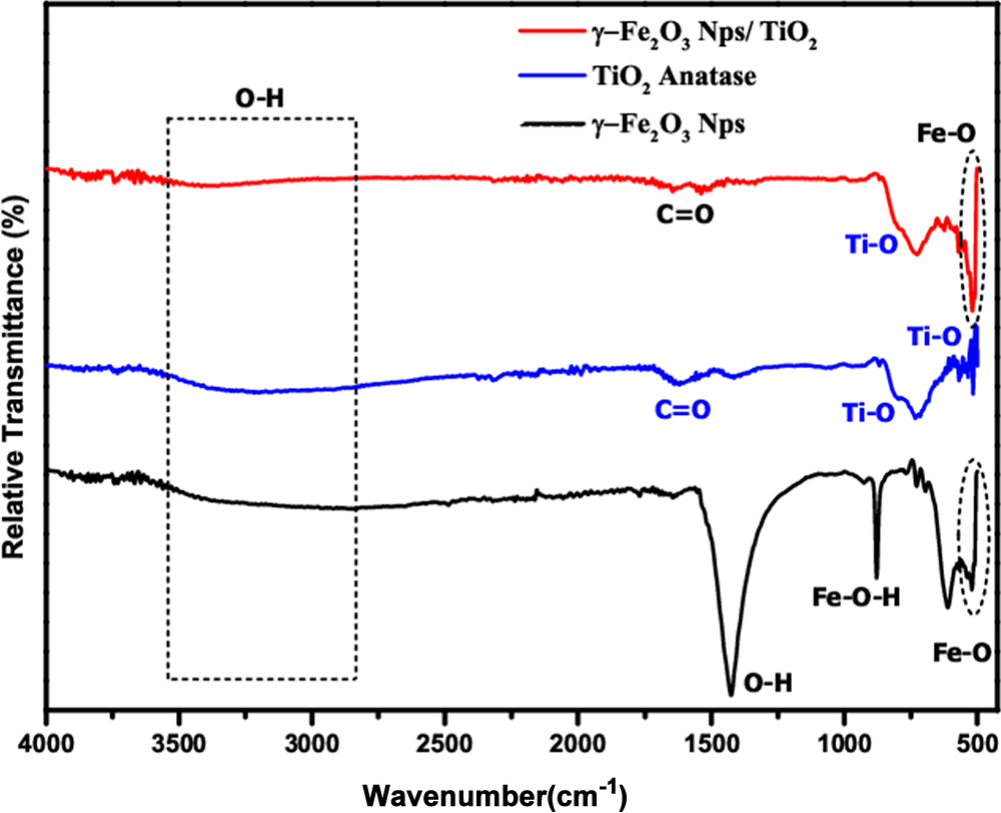
FTIR spectra of γ-Fe_2_O_3_ Nps, TiO_2_ anatase, and γ-Fe_2_O_3_ Np/TiO_2_ nanocomposites.

The FTIR spectra of γ-Fe_2_O_3_ Nps and the γ-Fe_2_O_3_ Np/TiO_2_ nanocomposites are illustrated in Figure 3.

FTIR spectrum of γ-Fe_2_O_3_ Nps (in black) shows two peaks at 520 and 611 cm^–1^, which were attributed to Fe–O stretching vibration, confirming the presence of iron oxide. The peak at 3400 cm^–1^ is related to the O–H vibration.^25,26^ Functional group vibration located at 877 cm^–1^ bands infers the hydroxyl groups (FeOOH) on the surface.^25,27^ The peak at 1426 cm^–1^ is related to the O–H elongation vibration band.^28^ In the FTIR spectrum (in red) for γ-Fe_2_O_3_ Nps/TiO_2_, the absorption band (3574–3000 cm^–1^) indicates the hydroxyl group (O–H) stretching vibration mode, and the intense peak at 520 cm^–1^ is related to Fe–O. In addition, the absorption peaks observed at 1630 and 1517 cm^–1^ were a result of the symmetric and asymmetric bending vibrations of the C=O bond.^29^ Also, the presence of an absorption band (625–875 cm^–1^) is related to the Ti–O and Ti–O–C vibrations.^17^

#### 3.1.3 XRD Characterization

**Figure 4:**
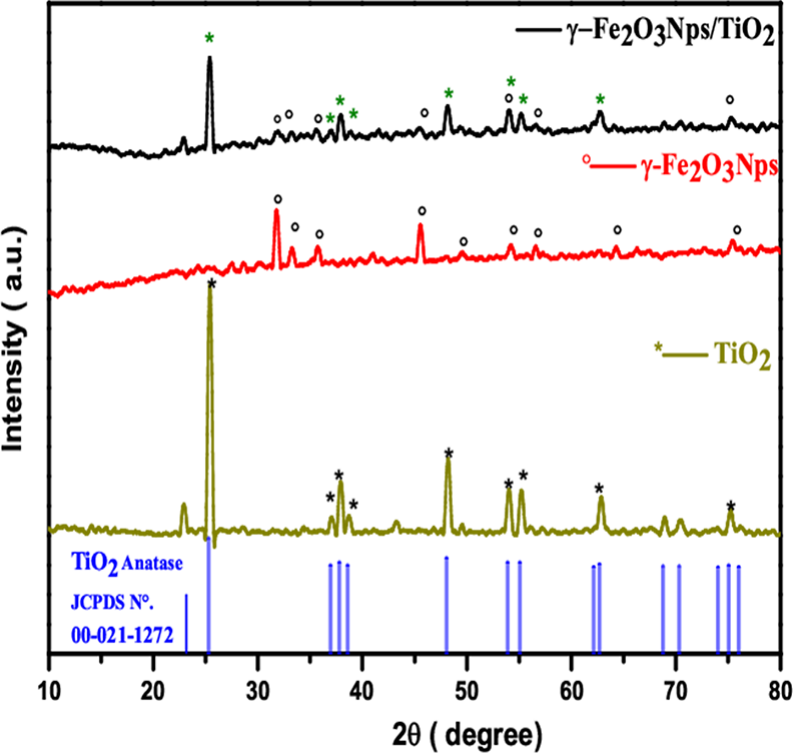
XRD pattern of standard TiO_2_ anatase (021–1272), TiO_2_, γ-Fe_2_O_3_ Nps, and γ-Fe_2_O_3_ Np/TiO_2_ nanocomposites.

Metals and metal nanocomposites were analyzed using powder XRD to determine the crystalline structure. Figure 4 shows the XRD patterns characterized by γ-Fe_2_O_3_ Nps, TiO_2_, and γ-Fe_2_O_3_ Np/TiO_2_ nanocomposites. The diffraction pattern obtained for the synthesized nanomaghemite γ-Fe_2_O_3_ shows diffraction peaks with 2θ = 31.85° (220); 35.63° (311); 45.54° (400); 49.52° (422); 54.21° (511); 56.66° (440); 72.9° (620); and 75.41° (533). These observed diffraction peaks can be associated with the cubic spinel structure, and all peaks are in good agreement with the diffraction patterns of the γ-Fe_2_O_3_ phase (JCPDS card 39–1346), which also corresponds to those of maghemite γ-Fe_2_O_3_ particles in the literature.^30,31^

A comparison of the XRD patterns of TiO_2_, γ-Fe_2_O_3_ Np, and γ-Fe_2_O_3_ Np/TiO_2_ composites is given in Figure 4. The phase of TiO_2_ was characterized by peaks at 2θ = 25, 38, 48, 54, 55, and 63°, indexed to the anatase (JCPDS No. 21–1272). For synthesized TiO_2_/γ-Fe_2_O_3_ composites, the peaks of the composite are relatively weaker than in the case of pure γ-Fe_2_O_3_ and TiO_2_; however, they match well with TiO_2_ and γ-Fe_2_O_3_ Np XRD patterns (Figure 3), which indicates that the final product is a complex composite of TiO_2_ and γ-Fe_2_O_3_ Nps. Also, the presence of distinct low-intensity peaks for γ-Fe_2_O_3_ Nps deposited on the TiO_2_ surface was observed, which might be due to the low concentration of deposited γ-Fe_2_O_3_ Nps.

The crystallite size of γ-Fe_2_O Nps is calculated from the following Scherrer’s eq 13.^32^
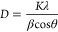
13where *K* is the shape factor (0.9); λ is the wavelength of CuK^α^ = 0.15418 nm; β is the fwhm in radians; and θ is the diffraction angle.

The peak at 2θ = 54.19 (311), which is the most characteristic peak of Fe_2_O_3_,^31^ was chosen to calculate *D*, and the size of the synthesized Nps was found to be 15 nm.

#### 3.1.4 SEM Characterization

**Figure 5:**
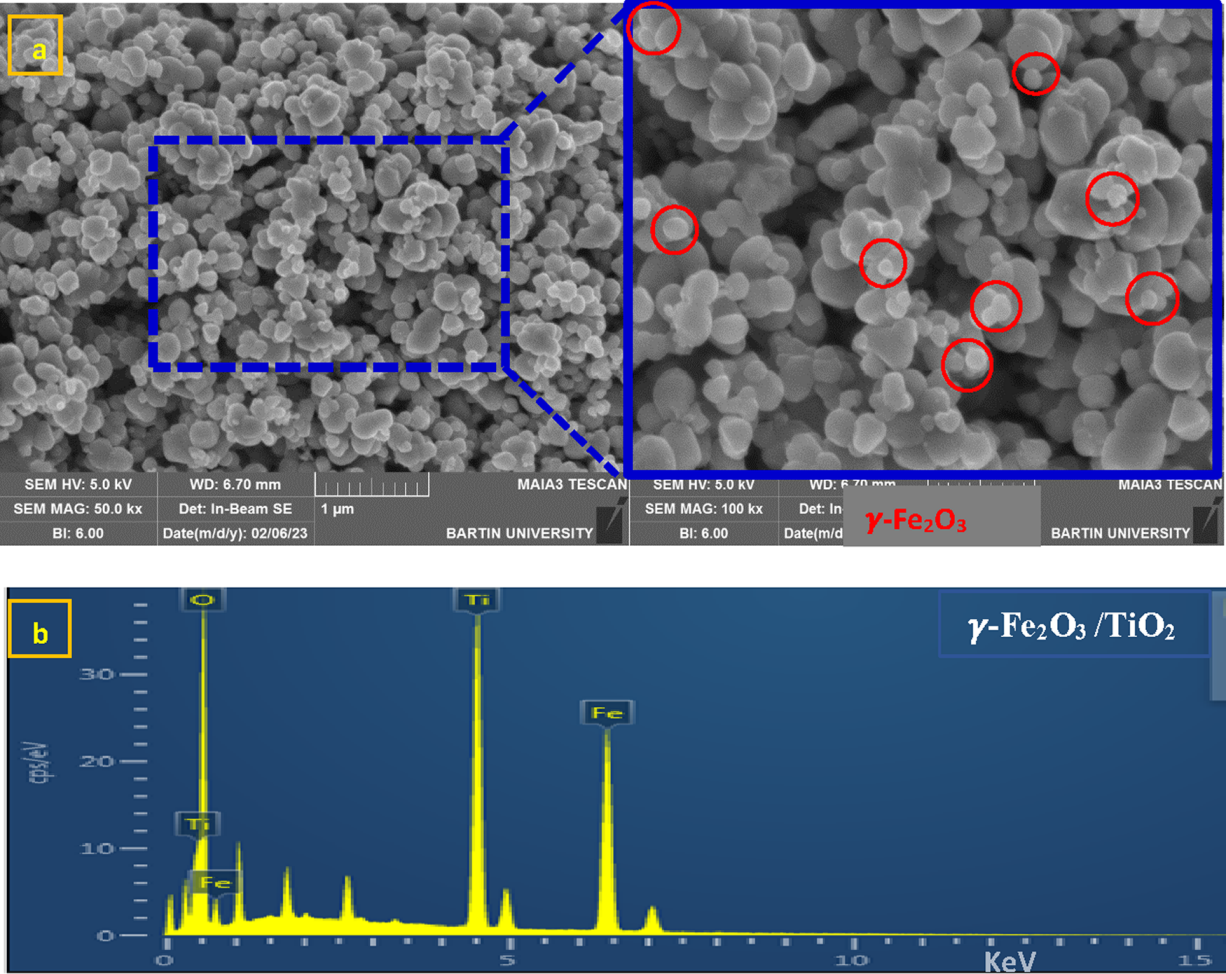
SEM micrographs (a) and EDS analysis (b) of γ-Fe_2_O_3_ Np/TiO_2_ nanocomposites.

Figure 5a shows SEM images of the γ-Fe_2_O_3_ Np/TiO_2_ bicomposite. The γ-Fe_2_O_3_ Np/TiO_2_ bicomposite consists of a significant number of clustered Nps that are constituted of smaller secondary Nps. The two types of Nps appear to have different shapes. The γ-Fe_2_O_3_ Nps resemble a diamond (nanopolyhedrons), whereas TiO_2_ resembles a sphere. Small molecules surround large molecules, and the smallest Nps have diameters of 40–80 nm. As a result, it is apparent that it is similar to the shape reported for C–Fe_2_O_3_/TiO_2_ nanoclusters used as a promising anode material for lithium-ion batteries.^33^ Furthermore, the EDS analysis of the γ-Fe_2_O_3_ Np/TiO_2_ nanocomposite (Figure 5b) shows O, Ti, and Fe elements, at 0.5, 4.5, and 6.4 keV, respectively. This analysis is consistent with the FTIR and XRD analysis results for γ-Fe_2_O_3_ Np/TiO_2_ nanocomposites.

### 3.2 Photocatalytic Degradation of OG under LED Light Irradiation

#### 3.2.1 Control Experiment

**Figure 6:**
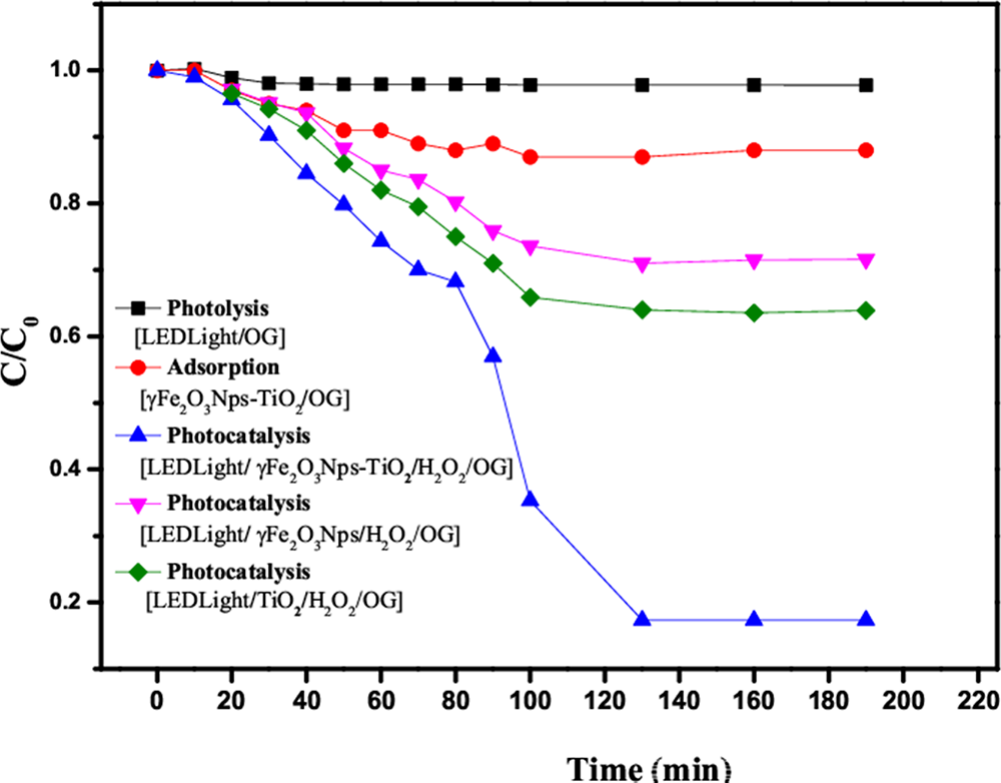
Experimental control of photolysis (LED-light/OG), adsorption (γ-Fe_2_O_3_ Nps-TiO_2_/OG), and the photocatalysis process using (γ-Fe_2_O_3_ Nps/TiO_2_, γ-Fe_2_O_3_ Nps, and TiO_2_).

As shown in Figure 6, a photolysis test was also carried out to look into the effect of the photocatalyst, and only 2.2% of removal efficiency of OG was achieved in the absence of the photocatalyst (γ-Fe_2_O_3_ Nps/TiO_2_) after 120 min, implying that the nanocatalyst plays a significant role in the removal of OG dye. The adsorption test was performed to study the effect of light on the removal efficiency of OG dye, and only 11.6% removal was achieved in 190 min, indicating that LED light plays an important role in the removal of OG dye. However, in the presence of LED light, nanocatalysts, and H_2_O_2_, the photodegradation efficiencies of γ-Fe_2_O_3_ Nps/TiO_2_, TiO2, and γ-Fe_2_O_3_ Nps were 84, 35, and 28%, respectively. This indicates that TiO_2_ decorated with nano-γ-Fe_2_O_3_ has a significantly higher photodegradation efficiency than γ-Fe_2_O_3_ Nps and TiO_2_ on the photodegradation of OG.

#### 3.2.2 Effect of Initial pH

**Figure 7:**
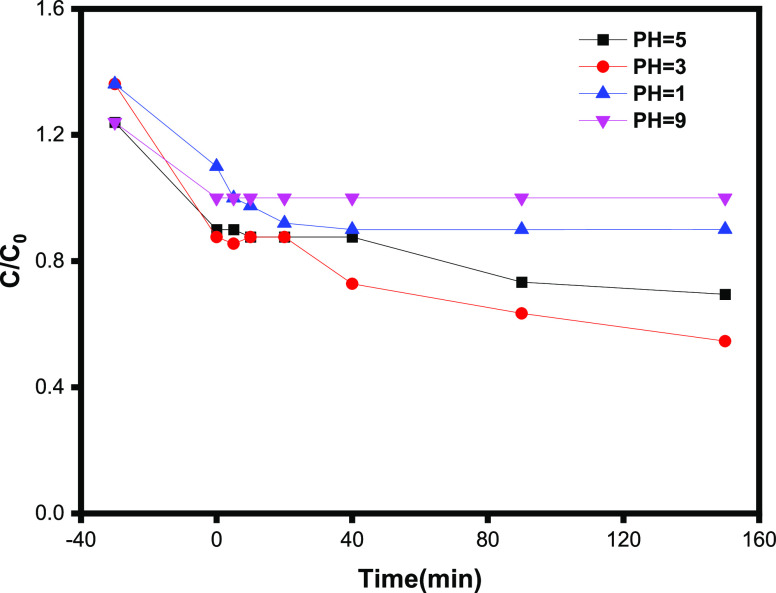
Effect of initial pH on photodegradation of OG under LED irradiation: [OG] = 5 mg L^–1^, [γ-Fe_2_O_3_ Nps/TiO_2_] = 0.8 g L^–1^_,_ and [H_2_O_2_] = 10^–1^ M.

In the pH range of 1.0 to 9.0, the impact of initial solution pH values on the decolorization of OG by the Fenton oxidation process was investigated. The findings are illustrated in Figure 7.

The initial pH had a direct influence on the decolorization of OG, with pH 3.0 providing the best decolorization efficiency. Photodegradation of OG was almost difficult to observe in 150 min of reaction at an initial pH of 1.0. It is primarily due to the formation of ferrous/ferric hydroxide complexes, which leads to the deactivation of the ferrous catalyst, resulting in a very small amount of ·OH being generated.^34,35^

On the other hand, when the initial pH is acidic (3.0 and 5.0), the decolorization efficiency of OG is significantly increased in 150 min. However, at an initial pH of 1.0, the decolorization efficiency of OG was reduced at highly acid pH conditions (pH < 3.0), and this could be explained by the formation of oxonium ions (i.e., H_3_O_2_^+^), which increased the stability of H_2_O_2_ and limited the generation of ·OH. Furthermore, the scavenging of ·OH by excess H^+^ is another reason for OG’s lower decolorization efficiency at pH 1.0.^35,36^ The synergism of adsorption and photocatalytic degradation may make OG degradation difficult at high pH (pH = 9.0). A suitable initial pH for the decolorization of OG by the Fenton oxidation process was recommended as 3.0 in this study. As a result, both high and low pH of solutions impact photocatalytic performance due to their influence on the surface charge and active sites of the photocatalyst. High pH can hinder optimal electron–hole pair generation and interactions with reactants, thus compromising overall photocatalytic efficiency. Also, in acidic solutions (pH < 3), the photodegradation of OG can be retarded by the high concentration of proton, resulting in lower degradation. An optimal pH of 3 can lead to the efficient adsorption of molecules of OG, a stimulus in charge transfer processes on catalytic active sites on the surface of γ-Fe_2_O_3_ Nps/TiO_2_, ultimately protecting the efficiency of the photocatalytic reaction.

#### 3.2.3 Effect of H_2_O_2_ Concentration

**Figure 8:**
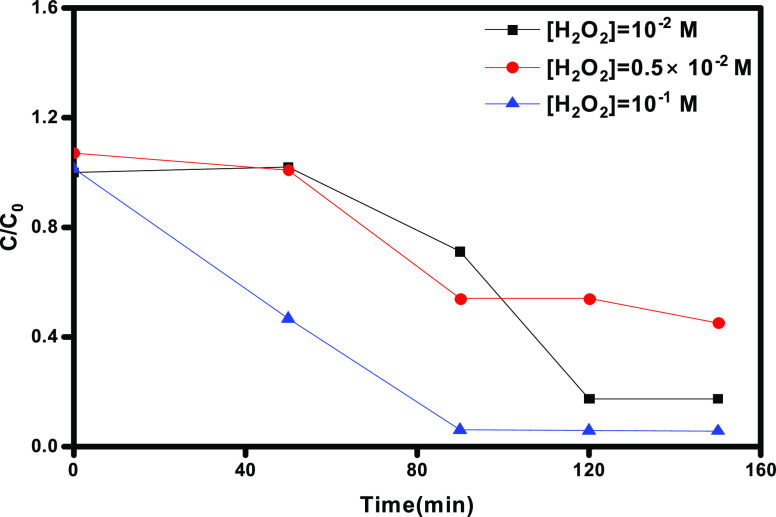
Effect of H_2_O_2_ concentration on photodegradation of OG: [OG] = 5 mg L^–1^, [γ-Fe_2_O_3_ Nps/TiO_2_] = 0.8 g L^–1^ and pH = 3.

The efficiency of the photodegradation of OG by the photo-Fenton catalytic process was tested at different H_2_O_2_ concentrations. Figure 8 shows the effect of the initial H_2_O_2_ concentration on the removal efficiencies of OG. As a result, with a concentration of 10^–2^ M of H_2_O_2_, 80% of OG degradation was obtained in 150 min. However, increasing the H_2_O_2_ concentration for 10^–1^ M in 150 min increased the degradation of OG to 98%, which could be due to the production of HO· radicals by H_2_O_2_ decomposition under LED light irradiation.

However, a low degradation efficiency (58%) was observed when a H_2_O_2_ concentration of 0.5 M was used, which could be attributed to the generation of hydroperoxyl radicals (HO_2_·), which are significantly less reactive (lower oxidation potential) than HO· radicals. It is a recognized fact that the introduction of hydrogen peroxide (H_2_O_2_) augments the efficiency of photocatalytic reactions by interacting with electrons (as indicated in eq 14). This interaction serves to mitigate recombination events and engenders an additional yield of hydroxyl radicals (HO·). Nonetheless, an excess of H_2_O_2_ is counterproductive due to its propensity to scavenge holes, resulting in the formation of hydroperoxyl radicals (HO_2_·) (eq 15) possessing a reduced oxidation potential compared to HO·:^37^
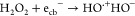
14
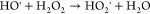
15

This explains why the degradation rate increases only until a limiting H_2_O_2_ concentration, which in our case is 10^–1^ M. Therefore, 10^–1^ M was considered an optimal H_2_O_2_ concentration for OG photodegradation experiments.

#### 3.2.4 Effect of the Amount of γ-Fe_2_O_3_ Nps/TiO_2_

**Figure 9:**
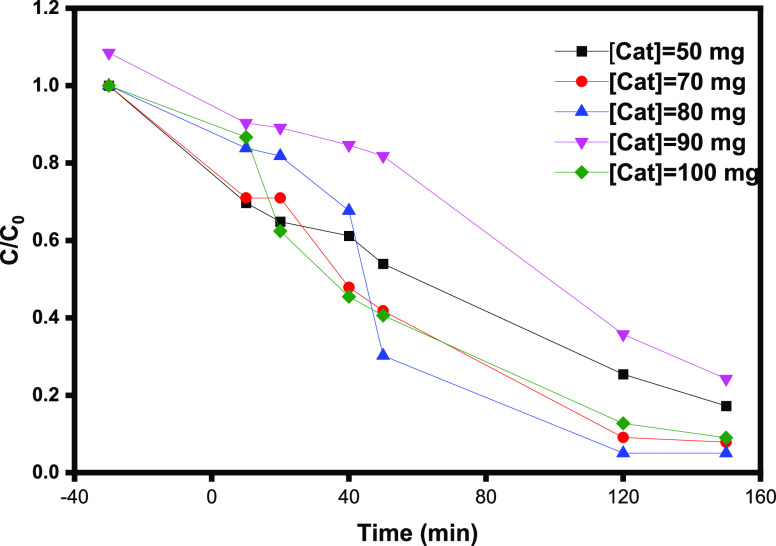
Effect of the γ-Fe_2_O_3_ Np/TiO_2_ amount on photodegradation of OG: [OG] = 5 mg L^–1^, [H_2_O_2_] = 1^–1^ M, and pH = 3.0.

The quantity of nanocomposites as nanocatalysts is another important parameter in assessing the effect of the photocatalyst process on dye removal efficiency.^38−42^ The effect of the photocatalyst γ-Fe_2_O_3_ Np/TiO_2_ nanocomposite amount on photodegradation of OG was investigated by varying the γ-Fe_2_O_3_ Np/TiO_2_ amount to 50, 70, 80, 90, and 100 mg in contact with 100 mL of OG solution (5 mg L^–1^), with same experimental conditions and under LED irradiation, and the obtained results are reported in Figure 9. It has been observed that the loading of photocatalysts has a significant impact on OG degradation. This might be due to an increase in the concentration of the γ-Fe_2_O_3_ Np/TiO_2_ catalyst promoting the reactive species available for OG degradation. We reported that the maximum degradation of the dye occurred at a photocatalyst dosage of 80 mg with 99.6% degradation, and for dosages of 50 and 70 mg had 82.5% degradation. However, at very high catalyst dosages (>80 mg), the turbidity of the suspension increases, reducing light penetration. As a result, there is an increase in light scattering, and the photodegradation process will be less effective. The ideal amount of the photocatalyst γ-Fe_2_O_3_ Np/TiO_2_ nanocomposite used in this study was 80 mg (0.8 g L^–1^) for the photodegradation process of the OG dye.

#### 3.2.5 Effect of Initial Concentration of OG Dye and Photodegradation Kinetics

**Figure 10:**
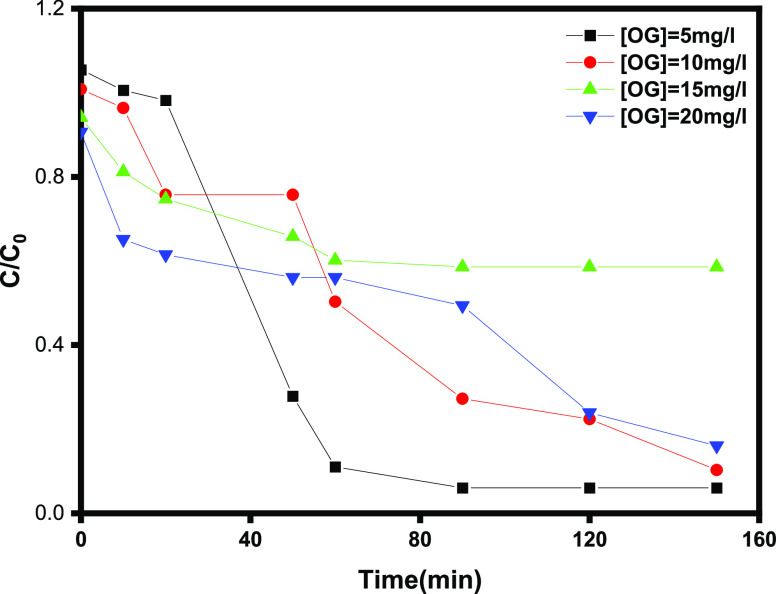
Effect of OG concentration on the photodegradation of OG under LED irradiation: [H_2_O_2_] = 10^–1^ M, [γ-Fe_2_O_3_ Nps/TiO_2_] = 0.8 g L^–1^, and pH = 3.

The photodegradation of various concentrations of OG was investigated, and the results are shown in Figure 10. It can be seen that the degradation efficiency of OG decreased as the concentration of OG increased. As the OG concentration increased from 5 to 20 mg L^–1^, the degradation efficiency of OG within 60 min of the reaction decreased from 95.0 to 40%. This is because an increasing concentration of OG results in a lower concentration of ·OH while maintaining the same dosages of H_2_O_2_, Fe^3+^/Fe^2+^, and Ti^4+^/Ti^3+^, resulting in a decrease in the OG degradation efficiency. The degradation efficiency was 90, 84, and 40.2% within 150 min when the OG concentration was, respectively, 10 mg L^–1^, 20 mg L^–1^, and 15 mg L^–1^. However, at an OG concentration of 5 mg L^–1^, the degradation efficiency reached 97.6% in just 90 min, under low irradiation energy (LED, 12 W). The initial OG dye concentration has a significant impact on the photodegradation mechanism in heterogeneous photocatalytic systems. A higher dye concentration reduces photodegradation efficiencies and reaction rates. Various organic dyes and catalysts have been studied for different classes of organic dyes, all supporting the statement above.^43^

The catalytic degradation efficiency reached more than 80% within 150 min under LED irradiation in the presence of the γ-Fe_2_O_3_ Np/TiO_2_ nanocomposite (Figure 10). The high degradation of OG dye, reaching a maximum removal efficiency of 97.6% (5 mg L^–1^) within a duration of 90 min, can potentially be attributed to the catalyst’s large surface area. Furthermore, the investigation of OG degradation data aimed to determine the reaction rate constant using the pseudo-first-order kinetic equation (Langmuir kinetic model) is as follows (eq 16):
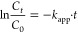
16where *k*_app_ is the pseudo-first-order rate constant and *C*_0_ and *C_t_* represent the OG concentrations at the initial and concentration after time “*t*”, respectively.

**Figure 11:**
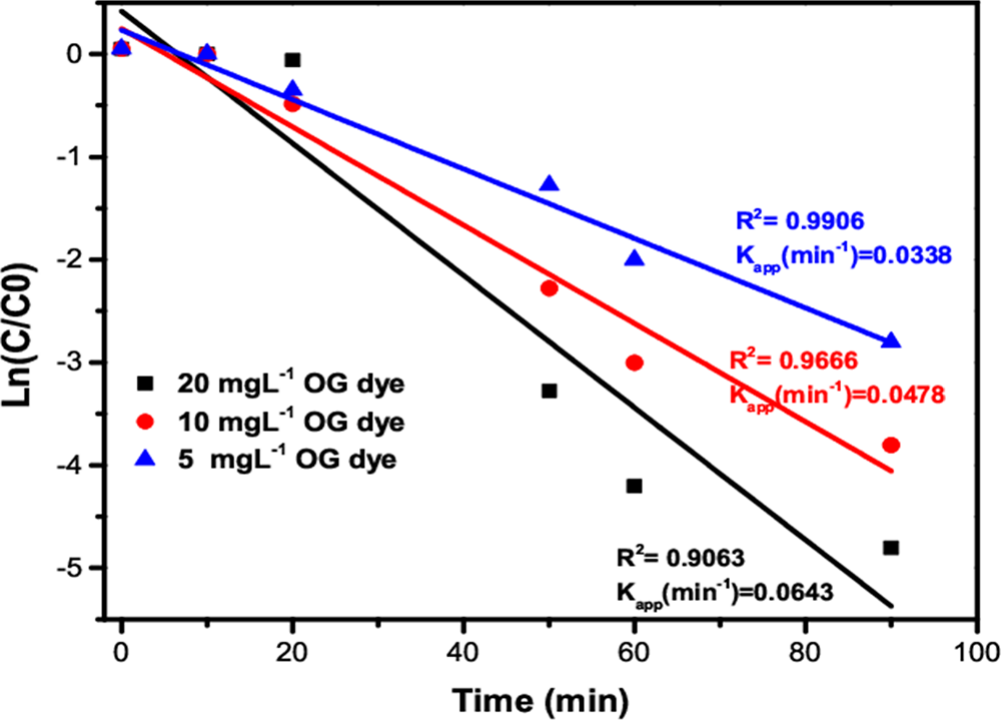
Kinetic analysis of OG photodegradation for different catalyst concentrations (5 to 20 mg L^–1^).

Figure 11 illustrates a linear relationship between ln (*C*_0_**/***C_t_*) and *t* within the concentration range of 5 to 20 mg/L, indicative of the pseudo-first-order kinetic model. The obtained results exhibit rate constants ranging (*k*_app_) from 0.0338 to 0.0643 min^–1^, with *R*^2^ values ranging from 0.90 to 0.99. These findings provide evidence that the photocatalytic decomposition of OG dye in an aqueous solution under LED light irradiation, utilizing the γ-Fe_2_O_3_ Np/TiO_2_ nanocomposite, can be accurately described by a pseudo-first-order kinetic model. Additionally, a similar investigation was carried out on NiZnAl layered double hydroxides as catalysts under solar irradiation for photocatalytic removal of OG.^44^

### 3.3 Mechanism of Photocatalytic Degradation of OG

Under eco-friendly LED white light irradiation, the action of the built-in electric field, and the concentration gradient, photogenerated electrons migrate from the conduction band of γ-Fe_2_O_3_ Nps to the conduction band of TiO_2_, yielding charge carriers, while photogenerated holes accumulate in the valence band of γ-Fe_2_O_3_ Nps. In addition, the synergistic effect of TiO_2_ and γ-Fe_2_O_3_ on the surface of the catalyst accelerates the redox reactions Fe^3+^/ Fe^2+^ and Ti^4+^/ Ti^3+^, resulting in an accelerated generation of the (·OH) radical. On the other hand, negative electrons in TiO_2_’s valence band react with oxygen dissolved in the dye solution to form superoxide anions and hydrogen peroxide.^45^ Thus, accumulated holes in γ-Fe_2_O_3_Nps’ valence band react with OH species on the catalyst’s surface to produce reactive hydroxyl radicals. Thus, (·OH) (or ·O^2–^) can react with the OG radical cation, causing degradation and, eventually, mineralized products.

Recently, the photodegradation of organic pollutants (dyes) in the Fe_2_O_3_/TiO_2_/H_2_O_2_ system has been the subject of new investigations. To understand the mechanism behind the photocatalytic degradation performance of dyes, these studies conducted radical trapping experiments to identify the radical species involved in the photocatalytic degradation of dyes within the Fe_2_O_3_/TiO_2_/H_2_O_2_ system (Fenton system). By employing BQ and TBA as scavengers for ·O^2–^, ·OOH, and ·OH radicals, the impact of these two quenchers on dye degradation was investigated, revealing that BQ and TBA can inhibit degradation efficiency. This suggests that ·O^2–^ and ·OH radicals are responsible for dye degradation.^46^ Additionally, a reduction in the percentage of dye degradation was observed upon the introduction of ascorbic acid into the photocatalytic reaction system. This observation demonstrates the significant role of superoxide radicals as active species in the dye degradation system.^47^ On the other hand, the presence of K_2_Cr_2_O_7_ (a scavenger for e^–^) and ascorbic acid (AA) was employed as potential scavengers for e^–^, h^+^, and ·O^2–^, leading to a deceleration in the degradation of NAP by the Fe_2_O_3_/TiO_2_ system under solar light exposure.^48^

**Figure 12:**
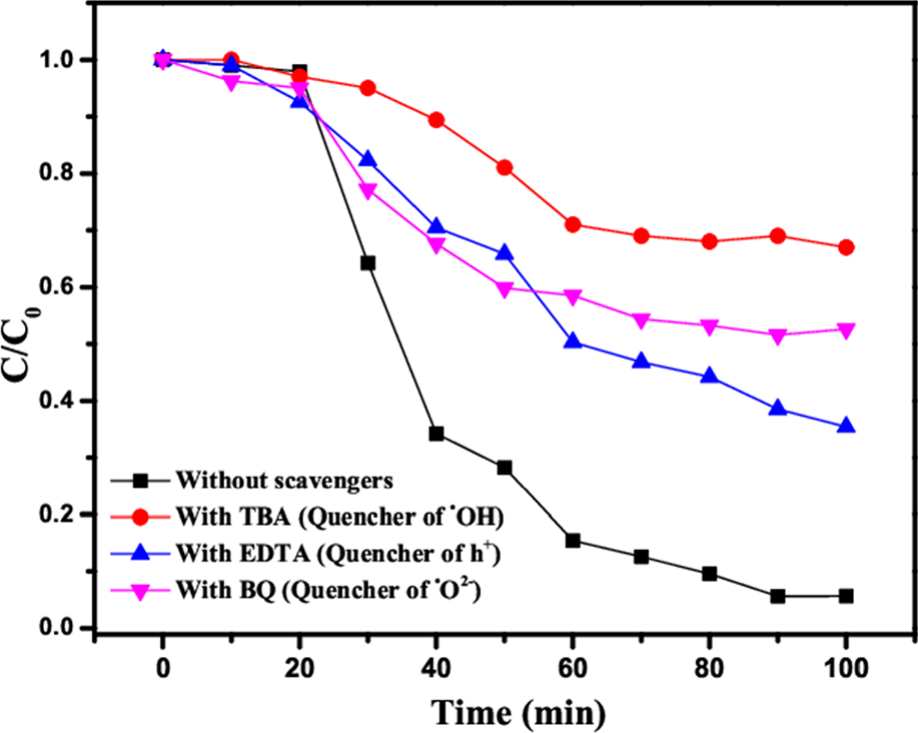
Trapping experiment of the active species during photocatalytic degradation of OG without added scavengers and with the addition of EDTA, BQ, and TBA, all under LED light irradiation.

To identify the radical species in the γ-Fe_2_O_3_ Np/TiO_2_-like photo-Fenton system during the photocatalytic degradation of OG, radical trapping experiments were conducted. For quenching tests, BQ, EDTA, and TBA were used as radical scavengers for ·O^2–^, h^+^, and ·OH radicals, respectively.^46−48^Figure 12 illustrates the inhibition experiments of the three quenchers on OG degradation in the γ-Fe_2_O_3_ Np-TiO_2_/H_2_O_2_ system. The addition of TBA and BQ rapidly inhibited OG degradation efficiency by approximately 32.8 and 49.5%, respectively. However, the addition of EDTA had little influence on the OG degradation (66%). Thus, this suggests that ·O^2–^ and ·OH are the primary active species and play a major role in the photocatalytic process, which is followed by electrons and holes (h^+^).

The reaction mechanism of the photocatalytic degradation of OG can be summarized as follows (eqs 17–26):
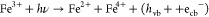
17
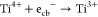
18
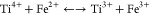
19
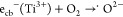
20
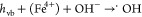
21
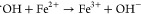
22
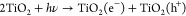
23
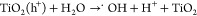
24
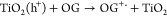
25
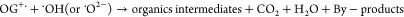
26

## 4 Conclusions

Currently, environmental issues have become a pressing concern in society due to water pollution caused by industrial effluents. Organic dyes have an important role to play in the detection of major organic pollutants in industrial wastewater. In this study, the γ-Fe_2_O_3_ Np/TiO_2_ nanocomposite material as a nanocatalyst has been prepared by the coprecipitation method. Characterizations and analysis methods confirmed that γ-Fe_2_O_3_ Nps deposited on the TiO_2_ surface, which exhibited low aggregation and abundant reaction sites. During the photodegradation process of dyes (OG as a model) under eco-LED-light irradiation, consideration was given to the effects of various experimental parameters such as reaction time, the dose of nanocatalysts, H_2_O_2_, and the initial amount of dye. The results indicate that the photodegradation efficiency of OG dye, catalyzed by Fe_2_O_3_ Nps/TiO_2_, reached 98 and 90% for initial dye concentrations of 5 and 10 mg/L, respectively, under the optimal reaction conditions: [H_2_O_2_] = 10^–1^ M, dose catalyst = 80 mg, pH = 3, *t* = 150 min, room temperature, and eco-white-LED irradiation. The pseudo-first-order model was used to represent the experimental kinetic data of OG dye degradation. Trapping experiments using different scavengers were conducted to determine the contributions of various radical species in the reactionary mechanism, showing that ·OH and ·O^2–^ radicals are predominant in the photodegradation of OG. The results of this study suggest that the γ-Fe_2_O_3_ Np/TiO_2_ nanocatalyst and LED irradiation have great potential for the photodegradation process of water contaminated with dyes. These results underscore the promising potential of our newly developed nanocatalyst in wastewater treatment.

## Data Availability

Not applicable.

## Notes

No animals or humans were used in the research.

## List of Abbreviations

Nps: nanoparticles
OG: Orange G
LED: light-emitting diode
UV–vis: ultraviolet–visible spectroscopy
FTIR: Fourier transforms infrared
XRD: X-ray analysis spectroscopy
SEM: scanning electron microscopy
